# Clinical Profile and Outcomes of Patients With Necrotizing Soft Tissue Infections: A Prospective Observational Study

**DOI:** 10.7759/cureus.87035

**Published:** 2025-06-30

**Authors:** Piyush Sharma, N Karthik, Mayank Badkur, Poonam Elhence, Sarika P Kobade, Pawan Garg, Ashok Puranik, Vaishali Paras, Sanket Kevadiya, Naveen Sharma

**Affiliations:** 1 General Surgery, All India Institute of Medical Sciences, Jodhpur, Jodhpur, IND; 2 Pathology, All India Institute of Medical Sciences, Jodhpur, Jodhpur, IND; 3 Microbiology, All India Institute of Medical Sciences, Jodhpur, Jodhpur, IND; 4 Diagnostic and Interventional Radiology, All India Institute of Medical Sciences, Jodhpur, Jodhpur, IND; 5 General Surgery, All India Institute of Medical Sciences, Guwahati, Guwahati, IND

**Keywords:** debridement, e coli, lrinec, mortality, nsti, postoperative outcomes

## Abstract

Introduction: Necrotising soft tissue infection (NSTI) is uncommon, and its management is complex due to its diverse clinical presentations, multiple associated comorbidities, and a wide range of potential microbial aetiologies. This study aims to illustrate the clinical profile, microbiological spectrum, and factors affecting mortality among patients with NSTI.

Methods: This single-centre, hospital-based, prospective observational study included all patients with NSTI aged 18 years or older. The primary outcome was the impact of time to surgery on mortality. Secondary outcomes included identifying the aetiology, microbiological flora, major co-morbidities, and overall outcomes in these patients.

Results: During the study period, 87 patients were enrolled. There were 65 (74.7%) male and 22(25.3%) female patients with an age range of 18 years to 88 years. Postoperatively, 18 patients succumbed to death, while 69 survived. Overall, the average timing of the first intervention after admission was six hours, with no significant statistical difference between the survivor and non-survivor group (p-value = 0.575). Our study found types I and II infections in 26 (34.5%) and 45 (65.4%) patients, respectively, and *Escherichia coli (E. coli*)* *was the most common isolate in both types. The antibiotic resistance pattern revealed increased resistance to third-generation cephalosporins and fluoroquinolones for the *Enterobacteriaceae *group and *E. coli*.

Conclusion: High APACHE II and LRINEC scores, anaemia, hypoalbuminemia, and high creatinine are associated with a higher risk of death. Prompt multidisciplinary management of these patients significantly improves outcomes and reduces mortality.

## Introduction

Necrotizing soft tissue infections (NSTIs) are rapidly progressive, life-threatening infections involving the skin and underlying soft tissues, characterized by extensive tissue necrosis, significant morbidity, high mortality rates, and substantial impact on patient outcomes and quality of life [1]. NSTI is uncommon; the incidence ranges between 0.3 and 15 cases per 100,000 people per year [2,3]. It often spreads along fascial planes, and in the early stages, the diagnosis can be challenging due to the absence of cutaneous manifestations [2]. However, patients often exhibit signs of systemic toxicity and experience pain that is disproportionately severe relative to the cutaneous findings. Several risk factors have been associated with NSTI, including advanced age over 75 years, diabetes, obesity, peripheral arterial disease, renal disease, tobacco dependence, alcohol dependence, substance abuse, immunosuppression, and soft tissue injuries [2,3].

NSTIs are among the few infections with high mortality rates of 6-33% and a significant risk of amputation, affecting even healthy young individuals [4]. Early diagnosis, resuscitation, appropriate antibiotics, and surgical debridement when necessary are all essential components [5]. Additionally, timely physiological support and critical care are crucial, preferably in an intensive care unit [6].

The management of NSTI is complex due to the diverse clinical presentations, multiple associated comorbidities, and a wide range of potential microbial aetiologies [7, 8]. Geographic differences in aetiology and microbiology have been shown nationally and regionally. Since NSTIs are relatively rare and often fatal, there are few studies in India [9, 10]. Variability in risk factors, environmental influence, exposures, and microbiological profile contributes to regional differences. No previous attempt has been made to characterize this entity in western India, particularly in Rajasthan. To identify the characteristics that distinguish it from the rest of the country, this study aims to describe the clinical profile, microbiological spectrum, and factors influencing mortality among patients with NSTI.

## Materials and methods

Study design and participants

This single-centre, hospital-based prospective observational study was conducted at the All India Institute of Medical Sciences, Jodhpur, a tertiary care facility in western India, from January 2021 to June 2022. The Institutional Ethics Committee approved the study (IEC/2021/3360). All patients with NSTI in the department of surgery were enrolled. Eligibility criteria included adults aged 18 years or older with NSTI initially diagnosed through clinical assessment and confirmed by operative findings or tissue diagnosis when necessary. Patients with diabetic foot and peripheral vascular disease associated with necrotizing fasciitis were also included. Patients with only cellulitis, abscess, and lymphedema were excluded.

Study procedure and outcomes

All the patients underwent a comprehensive assessment soon after reaching the emergency room. The patients were resuscitated and started on broad-spectrum intravenous antibiotics, later changed as per culture reports. The patients meeting the inclusion criteria were provided detailed explanations about the study using a patient information sheet, and written informed consent was obtained from willing participants. All cases with rapidly progressive soft tissue infection with tissue necrosis with or without systemic illness were included, and appropriate surgical interventions were performed. The diagnosis was confirmed based on operative findings of extensive soft tissue destruction extending beyond externally visible margins, non-adherent and friable fascia, dishwater toxic fluid, pus, and thrombosis of vessels. Excised tissue specimens were sent for microbiological evaluation after saline wash, and if required, for histopathologic evaluation. After the surgery, patients were transferred to wards or the ICU based on clinical status to provide organ and nutritional support. The wound was managed with daily dressing, and tissue viability was assessed daily. NPWT application was done as and when required based on wound status. Patients were discharged when satisfactory clinical status was achieved.

Scoring systems and classification used: Patients were assessed using the Laboratory Risk Indicator for Necrotising Fasciitis (LRINEC) and SIARI scores as predictive tools for necrotizing soft tissue infections, while the Acute Physiologic Assessment and Chronic Health Evaluation (APACHE) II score was used to estimate the risk of mortality [11-13]. NSTIs were classified according to Giuliano’s classification into four types based on microbiological aetiology [14].

The primary outcome was the impact of time to surgery on mortality among NSTI patients. Secondary outcomes included identifying the aetiology, microbiological flora, major co-morbidities, and overall outcomes in these patients. All the patients were followed up till discharge.

Sample size

This time-bound study included all NSTI patients, admitted to the surgery department of our institution, who met the eligibility criteria from January 2021 to June 2022.

Statistical analysis

Data were compiled and analyzed using the Statistical Package for the Social Sciences v. 29 (IBM Corp., Armonk, NY). Descriptive statistics were presented as percentages, means, and medians, as appropriate. Comparative analyses were conducted using the Pearson chi-square test, Fisher’s exact test, and the Mann-Whitney U-test. A p-value < 0.05 was considered statistically significant.

## Results

During the study period, 359 patients who presented to the emergency room were assessed for eligibility. Consequently, 87 patients diagnosed with NSTI were enrolled. There were 65 (74.7%) male and 22 (25.3%) female patients with an age range of 18 years to 88 years. Males were more commonly affected than females. There was no significant difference in the sex ratio between the survivor and non-survivor groups. There was no significant difference between survivor and non-survivor groups concerning the duration between the onset of symptoms and presentation (p-value = 0.960). The baseline characteristics of the study participants categorised by survival status are detailed in Table 1.

**Table 1:** Baseline characteristics of patients categorized by survival status *p-value calculated by Mann-Whitney U test; **p-value calculated by chi-square test; ***p-value calculated by Fisher’s exact test Abbrevations: IQR, interquartile range; PVD, peripheral vascular disease

Patient characteristics		Survivor group (n=69); n (%)	Non-survivor group (n=18); n (%)	p-value
Median age in years (IQR)		56 (45-68)	57 (46- 70)	0.42*
Sex (%)	Male	52 (80)	13 (20 %)	0.77**
	Female	17 (77.3)	05 (22.7 %)	0.77***
Median time to presentation in days (IQR)		3 (2-4)	3 (2-4)	0.960*
Site of involvement (%)	Head and neck	01 (1.4)	00 (0)	1.000***
	Upper limb	05 (7.2)	04 (22.2 %)	0.1546***
	Lower limb	45 (65.2)	08 (44.4 %)	0.1811**
	Thorax and abdomen	07 (10.1)	03 (16.7 %)	0.414**
	Perineum	11 (16.1)	03 (16.7 %)	1.000***
Comorbidities(%)	Diabetes mellitus	26 (37.7)	08 (44.4 %)	0.801**
	Hypertension	15 (21.7)	03 (16.7 %)	0.755***
	PVD	09 (13.0)	00 (0)	0.200***

Postoperatively, 18 (20.7%) patients succumbed to death, while 69 (79.3%) survived. Overall, the average timing of the first intervention after admission was six hours, with no significant statistical difference between the survivor and non-survivor group (p-value = 0.575). A comparison of predisposing factors and Giuliano's classification of NSTI is detailed in Table 2. The comparison of various preoperative laboratory parameters and clinical scoring systems associated with mortality is detailed in Table 3.

**Table 2:** Comparative analysis of time from admission to surgery and other outcomes *p-value calculated by Mann Whitney U test; **p-value calculated by Chi-square test ***p-value calculated by Fisher’s exact test # One survivor had a polymicrobial infection, including *Candida albicans*, which is also listed under the fungal category. Abbreviations: IQR, interquartile range; IV, intravenous; NSTI, necrotizing soft tissue infection

Outcome		Survivors (n=69); n (%)	Non-survivors (n=18); n (%)	Test statistic	p-value
Median time from admission to first surgery in hours (IQR)		5.9 (4.2-9.2)	6.1 (5-9.4)	U= 562.5	0.575*
Predisposing factors (%)	Trauma	09 (13.0 %)	03 (16.6 %)	_	_
	IV injection/ cannulation	01 (1.4 %)	01 (5.5 %)		
	Bite-animal/ insect/unknown	04 (5.8 %)	00 (0)		
	Thorn prick	02 (2.9 %)	01 (5.5 %)		
	Pre-existing soft tissue infection	04 (5.8 %)	00 (0)		
	Post-surgical intervention	01 (1.4 %)	00 (0)		
	Not-known	48 (69.6 %)	13 (72.2 %)		
Type of NSTI (%)	Polymicrobial	19 (27.5 %)	07 (38.9 %)	χ² = 0.73	0.392**
	Monomicrobial	36 (52.2 %)	09 (50 %)	χ² = 0.03	0.869**
	Fungal	01^# ^(1.4 %)	01 (5.5 %)	-	0.373***
	No growth	14 (20.3 %)	01 (5.5 %)	-	0.179***

**Table 3:** Comparison of preoperative clinical and laboratory parameters with mortality *p-value calculated by Mann-Whitney U test Abbreviations: IQR, interquartile range; TLC, total leukocyte count; APACHE II score, Acute Physiology and Chronic Health Evaluation II score; LRINEC score, Laboratory Risk Indicator for Necrotizing Fasciitis score; SIARI score, Surgical Intervention, Antibiotics, Resuscitation, and Intensive care score

Characteristic	Survivors (n=69)	Non-survivors (n=18)	Test statistic	p-value*
	Median (IQR)			
Preoperative haemoglobin (g/dL)	11.2 (9.7-13.1)	9.1 (7.1-9.8)	U = 303.5	0.0007
Preoperative TLC (×10³/cu mm)	13.7 (9.7-20.9)	16.6 (11.4-22.1)	U = 563.0	0.54
Preoperative creatinine (mg/dL)	1.0 (0.8-1.6)	1.5 (0.9-2.3)	U = 448.0	0.0384
Preoperative albumin (g/dL)	2.7 (2.3- 3.3)	2.0 (1.8-2.6)	U = 282.5	0.0002
APACHE II score at admission	6 (5-9)	10 (5.5–15.2)	U = 442.5	0.0366
LRINEC score at admission	4 (2-6)	6 (4–10.2)	U = 387.5	0.0046
SIARI score at admission	2 (1-5)	4 (2.7-5)	U = 477.0	0.0536

The antibiotic resistance pattern revealed increased resistance to third-generation cephalosporins and fluoroquinolones for the *Enterobacteriaceae* group and *E. coli*. The microbiological isolates according to Giuliano's classification of NSTI are listed in Table 4.

**Table 4:** Microbiological isolates in NSTI patients *One survivor had a polymicrobial infection, including *Candida albicans*, which is also listed under the fungal category. Abbreviations: NSTI, necrotizing soft tissue infection; MRSA, methicillin-resistant *Staphylococcus aureus*; MSSA: methicillin-susceptible *Staphylococcus aureus*

Isolate	Type I	Type II	Fungal
*Escherichia coli* (%)	11 (12.6)	14 (16.1)	-
*Acinetobacter baumannii *(%)	9 (10.3)	6 (6.9)	-
*Klebsiella pneumoniae* (%)	9 (10.3)	7 (8.0)	-
*Pseudomonas* (%)	7 (8.0)	4 (4.6)	-
*Enterobacter* (%)	8 (9.2)	1 (1.1)	-
*Proteus mirabilis *(%)	6 (6.9)	0 (0)	-
*Proteus vulgaris* (%)	0 (0)	1 (1.1)	-
*Morganella morganii* (%)	1 (1.1)	0 (0)	-
*Serratia marcescens* (%)	0 (0)	1 (1.1)	-
*Streptococcus pyogenes* (%)	4 (4.6)	4 (4.6)	-
MRSA (%)	4 (4.6)	5 (5.7)	-
MSSA (%)	0 (0)	2 (2.3)	-
*Candida albicans* (%)	1 (1.1)*	-	1 (1.1)*
Mucormycosis (%)	-	-	1 (1.1)

In our study, 31.8% of patients landed in amputation. Among survivors, definitive management of the raw area following recovery from NSTI included primary closure in five (5.7%), reconstructive wound coverage in 26 (29.8%), and healing by secondary intention in 19 (21.8%). A total of 21 patients (24.1%) underwent amputation. Non-survivors had a significantly greater incidence of intensive care unit admission, mechanical ventilation requirement, ventilator-associated pneumonia, and multi-organ dysfunction syndrome compared to survivors. A comparison of surgical management and postoperative outcomes between survivors and non-survivors is shown in Table 5. Additional details regarding clinical features stratified by outcome, as well as comprehensive antimicrobial sensitivity profiles of gram-negative and gram-positive isolates, are provided in Appendices 1-2.

**Table 5:** Comparison of Surgical Management and Outcomes *p-value calculated by chi-square test; **p-value calculated by Fisher’s exact test; ***p-value calculated by Mann-Whitney U test. Abbreviations: NPWT, negative pressure wound therapy; IQR, interquartile range; ICU: intensive care unit

Characteristic	Survivors (n=69)	Non-survivors (n=18)	Test statistic	p-value
Debridement (%)	66 (95.6)	18 (100)	χ² = 0.34	1*
NPWT (%)	22 (31.8)	05 (27.7)	χ² = 0.11	0.737*
Amputation (%)	19 (27.5)	02 (11.1)	-	0.146**
Median number of surgical procedures (IQR)	2 (1-3)	2 (1-4.25)	U = 507.0	0.303***
Median duration of postoperative hospital stay in days (IQR)	9 (7-15)	4 (2-12.3)	U = 378.5	0.007***
ICU admission (%)	3 (4.3)	15 (83.3)	-	<0.001**
Requirement of mechanical ventilation (%)	3 (4.3)	17 (94.4)	-	<0.001**

## Discussion

Several classification systems exist for NSTI, with one of the widely used being the bacteriological classification introduced by Giuliano et al. [14]. Type I infections are polymicrobial, involving a combination of gram-positive and negative bacteria and anaerobes. Type II infections are monomicrobial, primarily caused by group A β-haemolytic *Streptococcus* or *Clostridium* species. Type III results from gram-negative marine bacteria. Additionally, some researchers have identified a type IV infection associated with *Candida* and other fungal pathogens [15]. The incidence of type I and type II infections is generally comparable [16]. Type I infections are most commonly associated with *E. coli *and *Enterococcus species*, whereas type II infections are primarily caused by *Streptococcus pyogenes* and methicillin-resistant *Staphylococcus aureus* (MRSA) [2]. Our study found types I and II infections in 26 (34.5%) and 45 (65.4%) patients, respectively, and *E. coli *was the most common isolate in both types. Though polymicrobial growth is the most common cause in the literature, few studies have shown *E. coli* as the predominant cause [15, 16]. In our study, all isolates were sensitive to penicillin, and most showed sensitivity to ampicillin, vancomycin, and linezolid. Resistance was high for third-generation cephalosporins and fluoroquinolones in the *Enterobacteriaceae* group and *E. coli*. Since the majority of NSTIs are polymicrobial, the risk of the emergence of antibiotic resistance is higher. Moreover, the difference in mortality between type I and type II was not statistically significant in our study.

Though not an established cause of NSTI in isolation, *S. aureus* was not a major isolate in our series but remains a clinically important organism when present. Its ability to cause widespread disease is linked to various microbial characteristics. Notably, approximately 30% of the human population harbours it in the upper respiratory tract [17]. In addition, it produces many virulence factors that help in adherence, local and systemic spread, and host responses. Many strains of *S. aureus* have attained Panton-Valentine leukocidin (PVL) toxin-producing capabilities by exchanging PVL-producing phages [17]. Most of these strains are MRSA and are associated with severe cutaneous and soft tissue infections consistent with NSTI [17]. Antibiotic resistance is rampant in this group, and resistance to almost all relevant antibiotic classes has been reported. Most* S. aureus* strains produce penicillinase; this can be appreciated in our study, which shows high resistance to third-generation cephalosporin. All isolates were sensitive to linezolid. Most showed high sensitivity to vancomycin, gentamicin, and cotrimoxazole.

Menon et al. conducted a study in India, reporting pain as the most frequently reported complaint, followed by erythema or necrotic skin patches [10]. Our study revealed similar findings, and the pain was often disproportionately severe compared to the visible lesion, with a small necrotic skin patch frequently concealing the extensive underlying fasciitis

Despite advancements in understanding NSTI, the mortality rate remains substantial, ranging between 18% and 34% [18]. In the present study, a mortality rate of 18 (20.7%) was noted. A retrospective study conducted in India has documented a mortality rate of 25% [19]. The relatively lower mortality rate can be explained by the fact that the hospital in which the study was carried out is a tertiary facility in the region, where NSTI cases are managed routinely. Treating surgeons have developed a strong clinical suspicion for soft tissue infections, ensuring timely diagnosis and intervention.

In a retrospective study conducted in France in 2015, Hua et al. identified five independent risk factors linked to mortality in NSTI patients [7]. These factors included age over 75 years, multifocal NSTI, nosocomial infection, peripheral arterial disease, and the presence of severe sepsis or shock at the time of hospital admission. In other studies, factors associated with mortality included delayed first surgery, low body mass index, and deranged laboratory parameters such as total leucocyte count, haemoglobin, creatinine, sodium, and platelets [2, 8]. Wong et al. proposed the score Laboratory Risk Indicator for Necrotising Fasciitis (LRINEC), a predictive system for NSTI diagnosis. A score ≥6 is suspicious of NSTI, while ≥ 8 is strongly predictive and correlates with mortality risk [11]. However, the LRINEC score's positive predictive value varies between 57% and 92% [8,20]. Additionally, recent predictive scoring systems have been developed, including the SIARI score and the NSTI Assessment Score (NAS) [12, 21]. In our study, high APACHE II and LRINEC scores were associated with increased mortality risk. Also, non-survival was not associated with previously established risk factors like older age, higher urea, deranged coagulation profile, hyponatremia, and prolonged duration from admission to the initial surgical intervention compared with the survivor group in our study. However, parameters like low haemoglobin, low albumin, and high creatinine levels showed a significant difference between survival and non-survival groups. Anaemia, hypoalbuminemia, raised creatinine, high APACHE II and LRINEC scores, and the need for ICU admission and mechanical ventilation were associated with mortality in our study, and these findings are supported by the other studies [9, 10, 19, 22-23].

Surgical exploration and debridement play a vital role in diagnosing NSTI, assessing the severity of the infection, and achieving effective source control [21, 25]. Common findings consist of positive finger test, grey necrotic tissue, absence of bleeding, thrombosed blood vessels, dishwater-coloured pus, and non-contractile muscles [2]. Reducing the time from hospital admission to the initial debridement is essential for lowering mortality. A study found that patients who received operative intervention within the first 24 hours had a significantly higher survival rate compared to those who had surgery after 24 hours [19]. In 2016, Hadeed et al. reported a retrospective case series and concluded that surgical intervention within the first six hours after diagnosis of NSTI would improve hospital outcomes by shortening both the hospital stay and ICU stay [25]. In the present study, the median time from admission to the initial surgery was 6 hours (IQR: 4.3-9.4 hours), which was significantly shorter than the 19-35 hours reported in a previous study [1]. Similar to our study, there was no difference between survivor and mortality groups concerning the mean time between admission to first debridement in the study by Barupal et al. [26]. This difference may be attributed to our institution's status as a tertiary referral centre, where NSTI was often diagnosed at another facility before patient admission, and some degree of resuscitation had already been initiated. In addition to surgical debridement and antibiotics, organ support, antishock therapy, and multidisciplinary collaboration are effective modalities for reducing mortality [8,27]. In our setting, the admitting surgical department primarily manages the patient, and the intensivists treat patients with NSTIs on a call basis. Clinical, physiological, and laboratory parameters guide resuscitation. After the operation, patients would be shifted to the ICU based on clinical grounds to manage septic shock further and provide organ support.

Currently, few reports about NSTI exist in this region, and one such attempt, Barupal et al., performed a prospective observational trial in Udaipur, India [26]. It included 50 patients, most males (88%) with a mean age of 50.8 ± 17.1 years. Studies from other parts of India revealed similar demographics as in our study [9, 10, 19, 22, 23]. Barupal et al. found an identifiable cause in 72% of patients, with previous cutaneous sepsis (34%) being the most common, followed by trauma (30%). This was in contrast to our study, in which no predisposing cause was identified in 70% of cases. This is likely because our institution functions as a tertiary referral centre, and many patients were referred from peripheral health facilities without complete documentation of initial symptoms or triggering events such as minor trauma, skin infections, or insect bites. Furthermore, some patients presented late in the disease course when the primary lesion was no longer clinically evident. This highlights the aggressive and rapidly progressive nature of NSTI, where the initial cause may become obscure by the time of presentation. Similarly, studies from other parts of India reveal equivocal results [9, 10, 19, 22, 23]. Contrary to our results, the most common site was the perineum (64%), followed by the lower limb (14%) in the study by Barupal et al. Other studies support that the lower limb is the most common site, including the present study [9, 10, 19, 22, 23]. Diabetes mellitus was the most common comorbidity in our study, similar to other studies [10,27]. The most common isolate in the study, by Barupal et al., was *E. coli*, followed by *Klebsiella*, which is consistent with our results. Most studies from other parts of India have revealed a similar microbiological spectrum, with type II infection being far more common than polymicrobial infection, contrary to Western data [9, 10, 19, 22, 23].

Researching NSTI is challenging due to its rarity and clinical similarity to other soft tissue infections. Our study focused on patient characteristics and outcomes, omitting pathogen-related factors and host-pathogen interactions. Strengthening microbiological diagnostics in NSTI management is crucial, as survival in severe infections often correlates with identifying the causative organism and its antimicrobial resistance. However, assessing the prevalence and impact of specific microbes in NSTI remains difficult, as microbiological data in similar studies often lack precision. Additionally, this single-centre study faced limitations in determining the time between injury and hospital admission, relying instead on the duration from admission to the first operation. Our study did not capture the total time from symptom onset or time spent at peripheral centres before referral, which are likely critical contributors to outcomes. The lack of this data is a limitation, and it restricts our ability to fully assess the impact of the overall delay in surgical treatment. While our findings on delayed intervention were not statistically significant, the importance of timely surgical management remains well-established. This reinforces the need for early recognition and timely referral from primary and secondary centres to definitive care facilities.

## Conclusions

Our study highlights the unique clinical and microbiological landscape of NSTI in western India, emphasising the need for region-specific understanding and management protocols. Our findings underscore that patients presenting with high APACHE II and LRINEC scores, anaemia, hypoalbuminemia, and elevated creatinine levels are at a higher risk of mortality. These factors can serve as early indicators for aggressive management and prioritisation of care. Although the time from admission to surgical intervention was not statistically linked to mortality in this cohort, early surgical debridement remains a critical cornerstone in NSTI treatment. In addition to early diagnosis and rational use of antibiotics, multidisciplinary management of these patients significantly improves outcomes and reduces mortality. The study reinforces the importance of clinician awareness and vigilance in identifying NSTI early and highlights the need for robust microbiological surveillance to guide effective antimicrobial stewardship in managing these life-threatening infections.

## Figures and Tables

**Figure 1:**
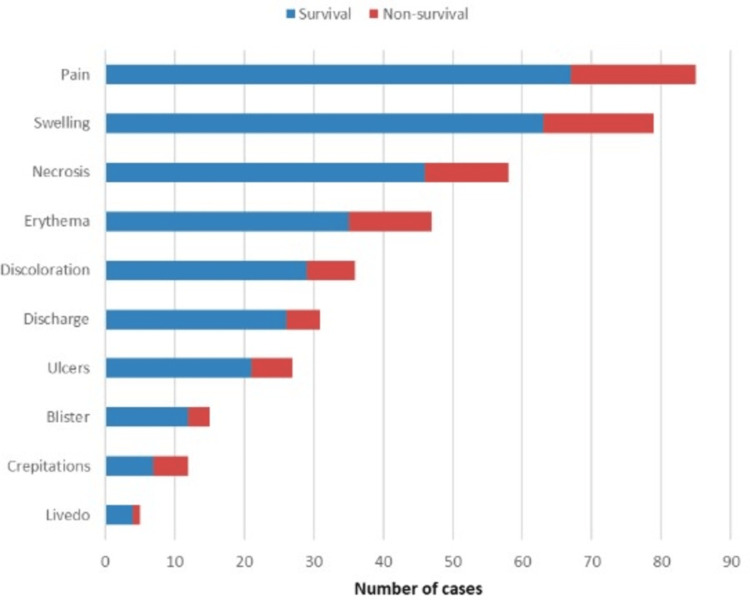
Clinical presentation The statement regarding disproportionate pain was based on clinical notes documented at presentation and is mentioned in the figure given above,  where we noted that many patients presented with severe pain, often exceeding the extent of visible cutaneous involvement.

## Appendices

Appendix 1

Details regarding clinical features stratified by outcome are provided in Figure 1.

Appendix 2

Details regarding comprehensive antimicrobial sensitivity profiles of gram-negative and gram-positive isolates are provided in Tables 6-7.

**Table 6:** Antimicrobial sensitivity of gram-negative isolates (n=84) Data are presented as percentage. E. coli, Escherichia coli. K. pneumoniae, Klebsiella pneumoniae. A. baumannii, Acinetobacter baumannii. E. cloacae, Enterobacter cloacae. P. aeruginosa, Pseudomonas aeruginosa. P. mirabilis, Proteus mirabilis. P. vulgaris, Proteus vulgaris. M. morganii. S. marcescens, Serratia marcescens

	E. coli N=24 (%)	K. pneumoniae n=16 (%)	E. cloacae N=9 (%)	P. mirabilis N=6 (%)	P. vulgaris N=1 (%)	M. morganii N=1 (%)	S. marcescens N=1 (%)	A. baumannii N=15 (%)	P. aeruginosa N=11 (%)
Piperacillin-tazobactum	16 (66.6)	7 (43.7)	5 (55.5)	4 (66.6)	1	1	1	2 (13.3)	8 (72.7)
Cefoxitin	7 (29.1)	1 (6.2)	0	4 (66.6)	0	1	0	0	0
Ceftriaxone	0	4 (25)	3 (33.3)	0	0	0	1	0	0
Ceftazidime-avibactum	0	3 (18.7)	.	0	0	0	0	1 (6.6)	4 (36.6)
Cefoperazone-sulbactum	13 (54.1)	3 (18.7)	5 (55.5)	3 (50)	0	1	1	1 (6.6)	2 (18.1)
Cefepime	4 (16.6)	4 (25)	5 (55.5)	1 (16.6)	0	1	1	.	6 (54.5)
Aztreonam	4 (16.6)	1 (6.2)	4 (44.4)	1 (16.6)	0	0	0	0	5 (45.4)
Imipenem	16 (66.6)	8 (50)	5 (55.5)	1 (16.6)	1	1	.	1 (6.6)	4 (36.3)
Meropenem	20 (83.3)	6 (37.5)	5 (55.5)	3 (50)	1	1	.	2 (13.3)	6 (54.5)
Ertapenem	4 (16.6)	1 (6.2)	0	0	0	0	0	0	1 (9.1)
Teicoplanin	.	.	1 (11.1)	.	.	.	.	.	.
Colistin	22 (91.6)	10 (62.5)	6 (66.6)	1 (16.6)	.	.	.	13 (86.6)	10 (90.9)
Linezolid	.	.	1 (11.1)	.	.	.	.	.	.
Gentamicin	14 (58.3)	2 (12.5)	7 (77.7)	5 (83.3)	1	0	0	3 (20)	2 (18.1)
Amikacin	19 (79.1)	5 (31.2)	7 (77.7)	3 (50)	1	1	0	3 (20)	5 (45.4)
Netilmicin	5 (20.8)	2 (12.5)	1 (11.1)	0	0	1	0	2 (13.3)	1 (9.1)
Tetracycline	2 (8.3)	.	.	.	.	.	.	.	.
Minocycline	0	3 (18.7)	2 (22.2)	0	0	0	0	4 (26.6)	1 (9.1)
Tigecycline	16 (66.6)	5 (31.2)	5 (55.5)	2 (33.3)	0	0	0	4 (26.6)	0
Cotrimoxazole	9 (37.5)	5 (31.2)	4 (44.4)	3 (50)	1	0	1	1 (6.6)	0
Ciprofloxacin	2 (8.3)	1 (6.2)	1 (11.1)	3 (50)	1	.	.	.	3 (27.2)
Levofloxacin	.	3 (18.7)	4 (44.4)	3 (50)	1	1	1	1 (6.6)	2 (18.1)

**Table 7:** Antimicrobial sensitivity of gram-positive isolates (n=20) Data are presented as percentages. MRSA: methicillin-resistant *Staphylococcus aureus*; MSSA: methicillin-sensitive *Staphylococcus aureus*

	*Streptococcus pyogenes*; n=9 (%)	MRSA; n=9 (%)	MSSA; n=2 (%)
Penicillin g	9 (100)	.	.
Ampicillin	7 (77.7)	.	.
Piperacillin-tazobactum	.	.	.
Cefoxitin	0	.	2 (100)
Cefuroxime	0	.	.
Cefotaxime	1 (11.1)	.	0
Ceftriaxone	3 (33.3)	0	0
Ceftazidime-avibactum	0	0	0
Cefoperazone-sulbactum	0	0	0
Cefepime	0	0	0
Aztreonam	0	.	.
Imipenem	0	1 (11.1)	.
Meropenem	0		1 (50)
Ertapenem	0	0	0
Vancomycin	6 (66.6)	7 (77.7)	2 (100)
Teicoplanin	1 (11.1)	8 (88.8)	1 (50)
Linezolid	6 (66.6)	9 (100)	2 (100)
Gentamicin	1 (11.1)	8 (88.8)	2 (100)
Amikacin	0	2 (22.2)	2 (100)
Netilmicin	0	1 (11.1)	.
Tetracycline	.	3 (33.3)	2 (100)
Tigecycline	.	8 (88.8)	2 (100)
Erythromycin	1 (11.1)	2 (22.2)	0
Clindamycin	6 (66.6)	1 (11.1)	1 (50)
Cotrimoxazole	0	8 (88.8)	2 (100)
Ciprofloxacin	3 (33.3)	3 (33.3)	1 (50)
Levofloxacin	3 (33.3)	2 (22.2)	1 (50)
Moxifloxacin	.	1 (11.1)	.
